# Distributions, Sources, and Backward Trajectories of Atmospheric Polycyclic Aromatic Hydrocarbons at Lake Small Baiyangdian, Northern China

**DOI:** 10.1100/2012/416321

**Published:** 2012-10-15

**Authors:** Ning Qin, Xiang-Zhen Kong, Ying Zhu, Wei He, Qi-Shuang He, Bin Yang, Hui-Ling Ou-Yang, Wen-Xiu Liu, Qing-Mei Wang, Fu-Liu Xu

**Affiliations:** MOE Laboratory for Earth Surface Processes, College of Urban and Environmental Sciences, Peking University, Beijing 100871, China

## Abstract

Air samples were collected seasonally at Lake Small Baiyangdian, a shallow lake in northern China, between October 2007 and September 2008. Gas phase, particulate phase and dust fall concentrations of polycyclic aromatic hydrocarbons (PAHs) were measured using a gas chromatograph-mass spectrometer (GC-MS). The distribution and partitioning of atmospheric PAHs were studied, and the major sources were identified; the backward trajectories of air masses starting from the center of Lake Small Baiyangdian were calculated for the entire year. The following results were obtained: (1) The total concentration of 16 priority controlled PAHs (PAH16) in the gas phase was 417.2 ± 299.8 ng*·*m^−3^, in the particulate phase was 150.9 ± 99.2 ng*·*m^−3^, and in dust fall was 6930.2 ± 3206.5 ng*·*g^−1^. (2) Vehicle emission, coal combustion, and biomass combustion were the major sources in the Small Baiyangdian atmosphere and accounted for 28.9%, 45.1% and 26.0% of the total PAHs, respectively. (3) Winter was dominated by relatively greater PAHs polluted northwesterly air mass pathways. Summer showed a dominant relatively clean southern pathway, whereas the trajectories in autumn and spring might be associated with high pollution from Shanxi or Henan province.

## 1. Introduction

 Polycyclic aromatic hydrocarbons (PAHs) are a group of compounds composed of two or more fused aromatic rings [1]. They have been of concern because of their potentially toxic, mutagenic, and carcinogenic properties [2–4]; therefore, 16 PAHs are included on the priority pollutants list of the US EPA [5]. Coal combustion, vehicle emission, the coking industry, and biomass burning are the main sources of PAHs [6]. They occur in the environment as complex mixtures of many components [7]. As one of the fastest growing countries in the world, China is suffering from severe contamination of PAHs from various sources that pose serious threats to ecosystems and human health [8].

The aquatic ecosystem is one of the major sinks of PAHs contamination [9], and the atmosphere plays an important role in the PAHs contamination of aquatic systems [10, 11]. PAHs exist in the ambient air as gases or adsorb to airborne particulate matter [12]. Therefore, the atmosphere is an important pathway for the transport of PAHs. Atmospheric deposition and diffusive exchange across the air-water interface are the major input routes for PAHs to the aquatic system [13]. It is necessary to study the pollution characteristics of atmospheric PAHs and identify the major emission sources to access the atmospheric influence of aquatic system.

**Figure 1:**
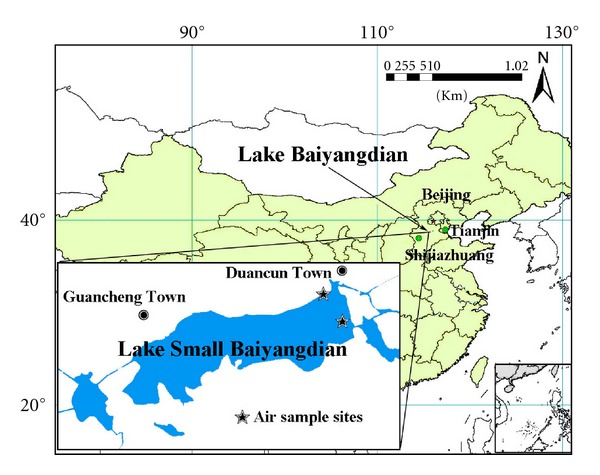
Location of Lake Small Baiyangdian and the sampling sites.

Lake Baiyangdian (38°43′~9°02′N/115°38′~116°7′E), with an area of 366 km^2^, is located in the triangular region defined by three large cities, Beijing, Tianjin, and Baoding (Figure 1).It is the largest freshwater lake in north China and is regarded as the “Pearl of North China.” Lake Baiyangdian plays an important role in maintaining the ecological balance of north China and in providing domestic, agricultural, and industrial water sources for the lake catchment [8]. The Lake Baiyangdian area is one of the areas most polluted by PAHs in China [8]. Understanding the distributions and sources of atmospheric PAHs in Lake Baiyangdian could be useful for the mitigation of PAHs pollution. Lake Baiyangdian is composed of 143 small lakes and ponds, among which Lake Small Baiyangdian is the largest, with a total area of 13.3 km^2^. This study of atmospheric PAHs at Lake Small Baiyangdian had four primary objectives: (1) to investigate the residual levels and spatial-temporal distributions of PAHs in gaseous and particulate phases and dust fall, (2) to elucidate the partitioning of PAHs in gaseous and particulate phases, (3) to identify major sources of PAHs, and (4) to analyze the backward trajectories of air masses starting from the center of Lake Small Baiyangdian.

## 2. Methodology

### 2.1. Measurement of PAHs in Gaseous and Particulate Phases and Dust Fall

#### 2.1.1. Sample Collection

The passive sampler designed by our laboratory [14] was used to collect gaseous and particulate PAHs samples from two sites located at the lakeside near the village and above the lake (Figure 1). A polyurethane foam (PUF) disk (110 mm diameter × 15 mm thick, density 0.024 g*·*cm^−3^) and a glass fiber filter (GFF, 110 mm diameter) were used as sampling media for gaseous and particulate phase PAHs, respectively. The PUF disks were previously extracted by Soxhlet with dichloromethane, acetone, and n-hexane sequentially for 12 h each reagent. The GFFs were preconditioned by heating in a furnace at 450°C for 4 h. After sampling, the PUF disks were wrapped with aluminum foil and kept in sealed bags sealed until measurement. The GFFs were wrapped with aluminum foil and saved in a desiccator without lights. The dust-fall samples were collected passively using stainless steel drums with a bottom area of 0.08 m^2^ that were placed next to the passive sampler. Before sampling, the drum bottom was covered with a mixed solution of 200 mL glycol and deionized water (V : V, 1 : 1) to prevent the decomposition of organochlorine pesticides. The dust fall in the drum was washed into a 1 L brown jar. Jars were dried at 400°C for 6 h prior to use. The gaseous, particulate, and dust-fall samples were collected during four periods: (a) October 7–November 14, 2007 (autumn); (b) November 15, 2007–March 15, 2008 (winter); (c) March 16–June 20, 2008 (spring); (d) June 21–September 5, 2008 (summer). The second period corresponded to the heating period in northern China.

#### 2.1.2. Extraction and Cleanup

The PUF, GFFs, and particle samples were extracted by Soxhlet with methylene chloride for 24 h, 12 h, and 24 h, respectively. The extract was concentrated to 1 mL, and the solvent exchanged with n-hexane and purified on a silica packed column. From the bottom to top, the column was filled with neutral silica gel (10 g, 3% deactivated) and anhydrous sodium sulfate (1 cm). The silica gel and anhydrous sodium sulfate were baked at 450°C for 4 h prior to use. The column was eluted with 50 mL of dichloromethane/hexane (2 : 3) at a rate of 2 mL·min⁡^−1^ to yield the PAHs fraction. The elute was concentrated on a rotary evaporator at a temperature below 38°C to approximate 1 mL, and internal standards (2-fluoro-1,1′-biphenyl and p-terphenyl-d14, 2.0 *μ*g·mL^−1^, J&K Chemical, USA) were added before samples were measured.

All sampling media were extracted immediately after harvesting. PUF chips and plugs were Soxhlet extracted in a 1 : 1 mixture of *n*-hexane and cyclohexane for 10 h. GFFs were extracted using the same procedure for 10 h. The extracts were concentrated by rotary evaporation to approximately 1 mL. Quantification was performed using the internal standard method using 2-fluoro-1,1′-biphenyl and *p*-terphenyl-*d*_14_ (2.0 *μ*g·mL^−1^; J&K Chemical, USA).

#### 2.1.3. Sample Analysis and Quality Control

All samples were analyzed on a gas chromatograph (Agilent GC6890/5973 MSD) connected to an HP-5MS capillary column and a mass selective detector (MSD, Agilent 5973). The column was programmed to warm from 60°C to 300°C over 5°C/min and then held isothermal for 20 min. The MSD was operated in electron impact mode at 70 eV, and the ion source temperature was 230°C. The mass spectra were recorded using selected ion monitoring mode.

Laboratory blanks and sample blanks were run with samples. Field blanks were sampling media (PUF and GFFs) taken with the samples. Both field blanks and laboratory blanks were extracted in the same way as the samples. All measurements were field-blank corrected. The mixed standard sample of 16 PAHs (PAH-Mixture, 610/525/550) produced by Chem Service Company was used to get the standard curve with the concentration series of 1 ppb, 10 ppb, 100 ppb, 1000 ppb. The blank experiment carried out using the glass beads to replace the gaseous and particular samples with the same extraction and purification procedures. Two procedure blanks were performed for about every eight samples, and the standard curve was calibrated using standard sample for about every 20 samples. The quantification was performed by the internal standard method using Nap-d8, Ace-d10, Ant-d10, Chr-d12, and Perylene-d12 (J&K Chemical, Beijing, China). The detection limits and method recoveries of the target PAHs are listed in Table 1.

#### 2.1.4. Calibration of the Passive Sampler

The following equation ([Disp-formula EEq1]) was used for the calibration of the gaseous and particulate phase PAHs in the air from the passive sampler to obtain the volume concentration, respectively. The equations were deduced by our colleagues based on the relationships between the gaseous and particulate PAHs from active and passive samplers in Hebei—the Beijing region where Lake Small Baiyangdian is located [15]:

(1) log⁡ PAHg(A)=0.7676  log⁡ PAHg(P) −2.167×10−9MWt3.776 +  1.6202,   (r2=0.878),PAHp(A)=PAHp(P)(e3.701−0.0314MWt), (r2=0.877),

where PAH_s_(A) and PAH_p_(A) represent the gaseous and particulate phase PAHs in air (ng·m^−3^) from the active sampler, respectively, PAH_s_(P) and PAH_p_(P) represent the gaseous and particulate phase PAHs in air (ng·d^−1^) from the passive sampler, respectively, and MWt represents the molecular weight of the PAH compound.

It should be pointed that there were some uncertainties associated with using the equations to estimate atmospheric concentrations and the implication for the analysis of ratios and PCA for source identification, although the sampling in the present study was performed within the area of the above equations produced, and the gas-phase sampling was similar particle size distributions.

### 2.2. Source Apportionment

The PAH isomer ratios method and the combined method of principal component analysis (PCA) and multiregression analysis (MLA) (PCA-MLA) were employed to identify the source of atmospheric PAHs at Lake Small Baiyangdian. The former method could provide only qualitative to semiquantitative results; however, the PCA-MLA method could present quantitative results.

#### 2.2.1. PAH Isomer Ratios

Isomer PAH ratios have been widely used to analyze atmospheric PAH sources [11, 16, 17]. Ratios of specific particulate phase PAHs are characteristic of different sources. Common ratios used include Fla/Pyr (mass 202), Baa/Chr (mass 228), and IcdP/BghiP (mass 276) [18].

For mass 202, fluoranthene to fluoranthene plus pyrene (Fla/Fla + Pyr), a ratio of 0.50 is usually defined as the petroleum/combustion transition point. For Fla/(Fla + Pyr), ratios between 0.40 and 0.50 are more characteristic of liquid fossil fuel (vehicle and crude oil) combustion, whereas ratios >0.50 are characteristic of grass, wood, or coal combustion; for mass 228, Baa/(Baa +Chr) ratios <0.20 indicate petroleum sources, from 0.20 to 0.35 indicate either petroleum or combustion, and >0.35 imply combustion; for IcdP/(IcdP + BghiP) ratios, <0.20 likely indicates petroleum, between 0.20 and 0.50 implies liquid fossil fuel (vehicle and crude oil) combustion, and ratios >0.50 imply grass, wood, and coal combustion [18].

#### 2.2.2. PCA-MLR Method

For quantitative investigations of possible sources of PAH contamination, principal component analysis (PCA) and multiregression analysis (MLR) were employed. PCA and MLR are common types of receptor models and have been successfully used for PAH apportionment [19]. PCA is a data reduction technique that aims to explain most of the variance in the data, while transforming a set of correlated measured variables into a set of a few uncorrelated components [20]. MLR is used to quantify the contribution of various source identified by PCA [4]. As a result of the PCA, normalized factor scores having a mean and standard deviation of 0 and 1 were obtained by PCA, then the factor scores from the PCA were used as independent variables, and the total PAH concentrations were used as dependent variable for a MLR.

The number of samples should be considered for PCA, a mathematical method widely used in principal component extraction in environmental statistics. The Kaiser-Meyer-Olkin (KMO) and Bartlett's test should be applied to analyze whether the samples were suitable for the PCA before it was used. When KMO >0.6, the data is suitable for PCA. When the number of samples is less than the number of variables, the KMO value cannot be calculated. However, this does not mean that the samples are not suitable for PCA. Some case studies showed that PCA could still be used to extract principal components, for instance, the atmosphere PAHs apportionment in Maryland [4], Lake Michigan [21], Prato [22], the sediment PAHs apportionment in the Pearl River Delta [23], and the soil PAHs apportionment in Delhi [24]. In the present study, the number of samples was eight, which is less than the number of variables; however, the PCA results showed that the samples were suitable for PCA (please see Section 3.2 for details), as indicated by previous case studies with the similar situation.

### 2.3. Backward Trajectory Analysis

The HYSPLIT (Hybrid Single-Particle Lagrangian Integrated Trajectory, Version 4.9) model [25] with NCEP/NCAR (National Centers for Environmental Prediction/National Center for Atmospheric Research) global reanalysis meteorological data was utilized to calculate 72 hours of backward trajectories starting from the center of Lake Small Baiyangdian (38.833°N, 115.937°E) at 00:00, 06:00, 12:00 and 18:00 UTC each day. The receptor height was set at 200 m as the lower level of the atmospheric boundary layer.

It has been demonstrated that clusters of trajectories arriving at a receptor location can serve as surrogates for different synoptic circulation patterns [26]. The nonhierarchical clustering algorithm (k-means) on the basis of the Euclidean distance is a common method of classifying air trajectories into subsets [26–28]. The optimum number of clusters was determined by comparing and analyzing the *R* ^2^ statistics [29] with the number of clusters included in the analysis. *R* ^2^ is defined as the proportion of the variance explained by the current number of clusters. A significantly large change in *R* ^2^ indicates that two highly dissimilar clusters are aggregated. Borge et al. [30] proposed a two-stage clustering procedure to further investigate “short” trajectory clusters with unclear directionalities. This method has been applied by Zhu et al. [31] to study the transport pathways and potential sources of PM_10_ in Beijing. In this study, the two-stage method was applied during the spring period because it was found that many short trajectories were grouped together in the spring, although they came from heterogeneous regions.

## 3. Results and Discussion

### 3.1. Residue Levels and Seasonal-Spatial Distributions of Atmospheric PAHs

#### 3.1.1. Residue Levels of Atmospheric PAHs

The residue levels of PAHs in the gas-phase, particle-phase, and dust fall in the air were presented in Table 2. The PAH16 concentrations in the gaseous phase varied from 84.4 to 982.6 ng·m^−3^, and the average concentration was 417.2 ± 299.8 ng·m^−3^. PAH16 in particulates varied from 84.4 to 982.6 ng·m^−3^, with a mean value of 150.9 ± 99.2 ng·m^−3^. PAH16 in the Baiyangdian atmosphere (gas+particle) ranged from 104.2 to 1250. 2 ng·m^−3^, with a mean value of 548.6 ± 392.4 ng·m^−3^. The dust PAH16 concentration in this study ranged from 2916.9 to 12387.2 ng·g^−1^, and the mean value was 6930.2 ± 3206.5 ng·g^−1^.

From Table 2, we can see that the PAHs compositions were quite different in the gas, particulate, and dust fall samples. The PAHs in the gaseous phase were dominated by LMW-PAHs (93.5%); MMW-PAHs and HMW-PAHs accounted for 6.2% and 0.5% of the total PAHs, respectively. However, in the particulate samples, the proportion of LMW, MMW, and HMW PAHs were more balanced, accounting for 37.8%, 38.2%, and 33.7%, respectively. In the dust fall samples, the percentages of LMW, MMW, and HMW PAHs were 50.2%, 38.2%, and 11.6%, respectively.

#### 3.1.2. Seasonal-Spatial Distributions of Atmospheric PAHs

**Figure 2:**
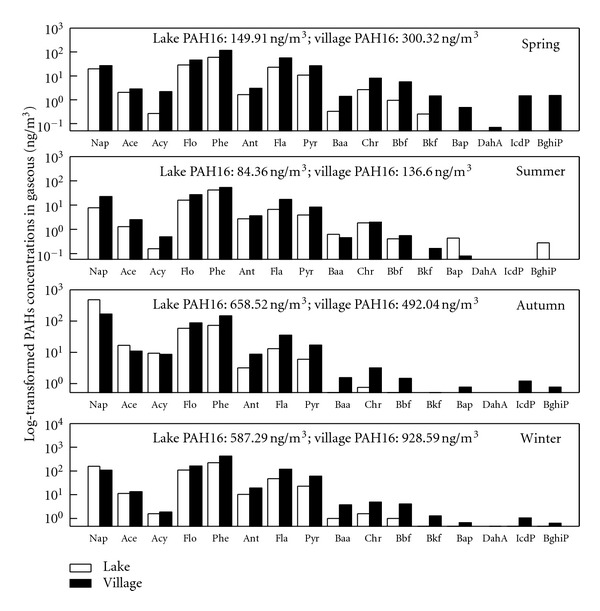
Seasonal-spatial variation in PAHs in the gaseous phase at Lake Small Baiyangdian.

**Figure 3:**
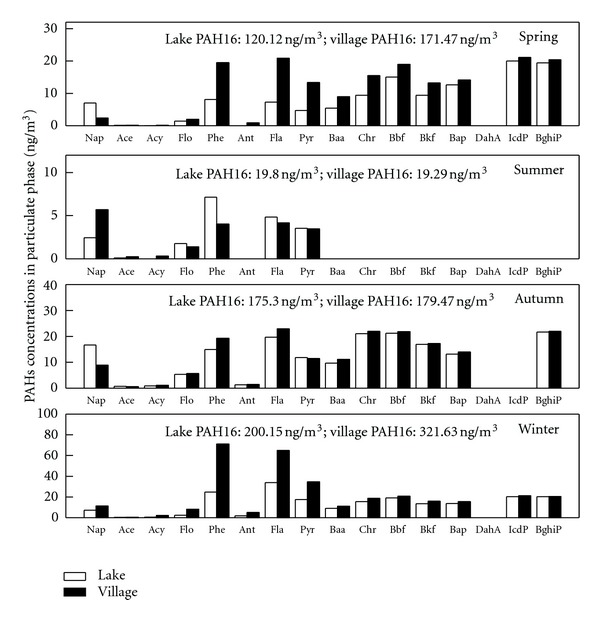
Seasonal-spatial variation in PAHs in the particulate phase at Lake Small Baiyangdian.

**Figure 4:**
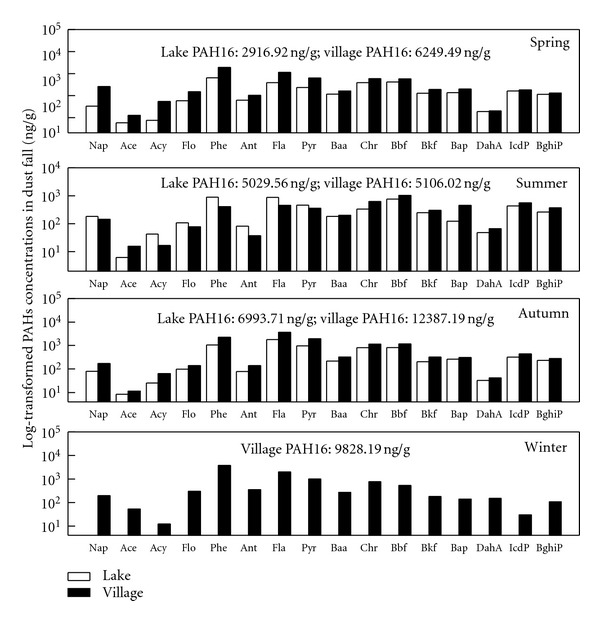
Seasonal-spatial variation in PAHs in the dust fall at Lake Small Baiyangdian.

The seasonal-spatial distributions of the PAHs in the gas-phase, particle-phase, and dust fall are shown in Figures 2, 3, and 4, respectively. The PAHs in the gaseous phase and particulate phase followed similar seasonal trends. The residual levels of PAH16 in the seasonal gas-phase contents exhibited the following order from high to low values : winter (757.94 ng·m^−3^) > autumn (575.33 ng·m^−3^) > spring (225.11 ng·m^−3^) > summer (110.48 ng·m^−3^). The seasonal particulate samples were ranked in the same order: winter (260.89 ng·m^−3^) > autumn (177.38 ng·m^−3^) > spring (145.79 ng·m^−3^) > summer (19.55 ng·m^−3^). In the dust fall, the seasonal average concentrations from high to low were winter (9.83 *μ*g·g^−1^) > autumn (9.69 *μ*g·g^−1^) > summer(5.07 *μ*g·g^−1^) > spring (4.58 *μ*g·g^−1^).

The average annual gaseous PAH16 content at the lake site was 370.0 ± 294.7 ng·m^−3^ and at the village site was 464.39 ± 341.9 ng·m^−3^; the particulate phase content was 128.8 ± 80.0 ng·m^−3^ at the lake site and 173.0 ± 123.5 ng·m^−3^ at the village site. A one-way ANOVA was employed to compare the spatial differences. The results showed no significant differences between the gaseous (*P* = 0.62) and particle phases (*P* = 0.19); however, a difference at a significant level of 0.01 was found between the lake and village sites for the dust samples. The dust fall PAHs concentration at the lake site was 4980.1 ± 2038.8 ng·g^−1^, which was much lower than the content at the village site (8392.7 ± 3337.3 ng·g^−1^). This suggests that human activities (e.g., cooking) have a significant effect on the PAHs contents of dust fall.

PAHs atmospheric residual levels in this study were compared with other research within and outside of China. Generally, PAH content in this study was at the same level or lower than most research in China but much higher than most reports from other countries. PAH content at Lake Small Baiyangdian was somewhat greater than found in Guangzhou (313 ng·m^−3^+23.7 ng·m^−3^) in southern China [32] and only slightly less than found in Tianjin in eastern China (485 ng·m^−3^+267 ng·m^−3^) [33]. The concentrations at Lake Small Baiyangdian were much greater than reported in Izmir, Turkey (PAH15 average 25.2 ng·m^−3^ in summer and 44.1 ng·m^−3^in winter)[34], Southern Chesapeake Bay (total 5.31–71.6 ng·m^−3^) [35], or Athens, Greece (4.8-76 ng·m^−3^) [36]. The concentrations at Lake Small Baiyangdian were also greater than found in lake samples in Chicago (PAH14, 92.3–244.9 ng·m^−3^) [12], which were considered to be highly contaminated.

#### 3.1.3. Relationships of PAH Distributions in the Gaseous and Particulate Phase

**Figure 5:**
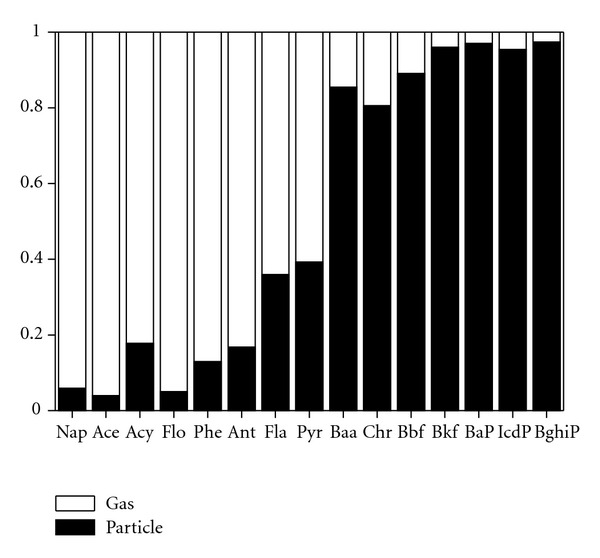
Ratio of particulate and gaseous PAHs (P/G) at Lake Small Baiyangdian.

Equation (2) was used to calculate the particle-gas (P/G) ratios of 16 PAHs between the particulate and gaseous phases:

(2) Gp=CparticlesCgas,,

where *C*_particle_ (ng·m^−3^) was the PAH concentration in the particle phase and *C*_gas_ (ng·m^−3^) was the concentration in the gas phase. The partition was shown in Figure 5. Only data points with concentrations above the detection limit for both phases were included in the calculations. The particulate-gas partition ratios varied from 0.48 (Ace, autumn lake) to 3738.65 (Phe, winter village). From the figure, it appears that low molecular weight PAHs were largely contained in the gaseous phase, whereas high molecular weight PAHs were distributed in particulates.

**Figure 6:**
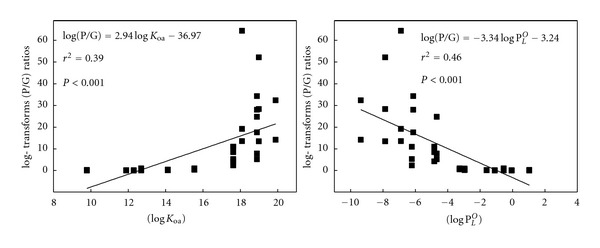
Relationships between log (P/G) and log⁡⁡*K*_Oa_ as well as log⁡ P_L_^O^ at Lake Small Baiyangdian.

Much work has been done to investigate the factors influencing the G/P ratios, the subcooled liquid vapor pressure (log⁡ P_L_^O^), and the octanol-air partition coefficient *K*_oa_ [37, 38]. Correlation analysis and linear regression were used to detect the relationship between *K*_oa_, P_L_^O^ and G/P ratios of PAHs, as shown in Figure 6. Significant correlations (*P* < 0.01) were found between G/P ratios and *K*_oa_ (*P* < 0.01), P_L_^O^ (*P* < 0.01). In this study, the following relationship were found:

(3) log⁡ ⁡(PG)=2.941log⁡⁡KOa−36.97,log⁡ (PG)=3.341log⁡⁡PLO−3.24.

This result suggested that *K*_oa_,  *P*_L_^O^ values have a significant influence on the partition of PAHs between the gas and particle phases, such that PAHs with higher *K*_oa_ but lower P_L_^O^ values are more easily absorbed onto particles.

### 3.2. Source Apportionment of Atmospheric PAHs

**Figure 7:**
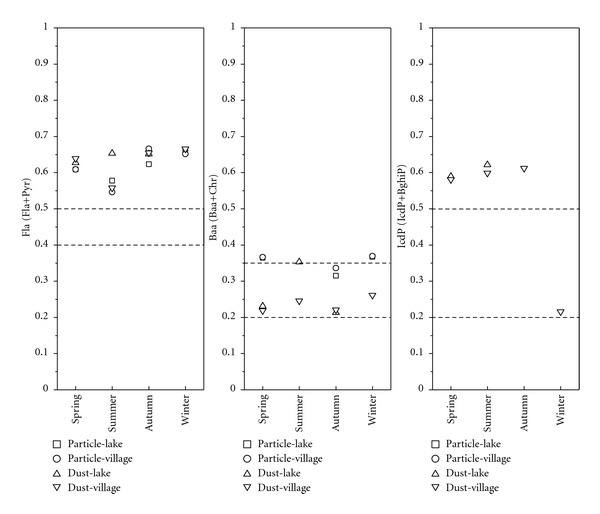
Isomer ratios of (Fla/Fla + Pyr), Baa/(Baa + Chr), and IcdP/(IcdP + BghiP).

The ratios of Fla/(Fla + Pyr) and IcdP/(IcdP + BghiP) are presented in Figure 7. From the figure, we can see that most of the ratios of Fla/(Fla + Pyr) were greater than 0.5. The values of IcdP/(IcdP + BghiP) were near the transition line of 0.5, with five points above the line, two points near the line, and another point between 0.2 and 0.5. Most of the Baa/(Baa + Chr) values were between 0.2 and 0.35. The results of Fla/(Fla + Pyr) and IcdP/(IcdP + BghiP) indicated that the sources of PAHs were primarily grass, wood, or coal combustion. However, the results of IcdP/(IcdP+BghiP) also implied that liquid fossil fuel combustion was also very important; therefore, the ratio points were near the 0.5 line. In contrast, the values of Baa/(Baa + Chr) implied that the PAHs at Lake Small Baiyangdian came from either petroleum or combustion or even a mixture of them. The high values of Fla/(Fla + Pyr) correspond with the results of IcdP/(IcdP + BghiP) and support the conclusion that combustion was the most important source of PAHs at Lake Small Baiyangdian, although the results of IcdP/(IcdP + BghiP) and Baa/(Baa+Chr) implied that liquid fossil fuel combustion might also exist in the area.

Using PCA, three components were extracted that represented more than 90 percent of the total variance. The rotated component matrix of the gas and particulate phase PAHs at Lake Small Baiyangdian is shown in Table 3. The primary sources of vehicle emission, coal combustion, and biomass combustion were identified. Factor 1 accounted for 38.86% of the total variance and was heavily weighted by Baa, Chr, Bbf, Bkf, Bap, IcdP, and BghiP. BghiP has been identified as tracers of autoemissions [39, 40]. Elevated levels of Bkf relative to other PAHs have been suggested to indicate diesel vehicles. IcdP was also found in both diesel and gas engine emissions [4], and this source appeared to be vehicle emission. The second factor accounted for 36.67% of the total variance. Factor 2 was predominately weighted by Ant Phe Flo Fla, Pyr [11, 41]. According to the literature, Flo, Pyr, Phe, and Ant were considered to be predominantly coal combustion profiles [4], and factor 2 suggests a coal combustion source. Factor 3 was heavily weighed by LMW PAHs, such as Nap, Ace, and Acy, and accounted for 21.42% of the total variance. PAHs produced by wood combustion are predominately low molecule weight PAHs [42, 43]. Acy and Phe are mentioned as markers for wood combustion [2]. Therefore, Factor 3 indicates a biomass combustion source.

The factor scores from the PCA and the total PAH concentrations were used as independent and dependent variable, respectively, for an MLR, and the following equation was obtained:

(4) ∑(gas+particles  PAHs)=0.480σPAHFS1+0.749σPAHFS2+0.432σPAHFS3.

The percentages of the sources from vehicle emission, coal combustion, and biomass combustion could be calculated (8). The results showed that the sources from vehicle emission and coal and biomass combustions accounted for 28.9%, 45.1%, and 26.0% of the total PAHs, respectively. Coal was widely used in northern China to produce energy. In 2007, 695000 tons of coals were burned in China, accounting for 69.5% of the proportion of total energy consumption; therefore, coal combustion made the largest contribution to the atmospheric PAHs. Although biomass was used to a lesser extent than coal for energy production, biomass still represented a much greater contribution to the PAHs in the village, especially in cooking and heating. We also found that wood or coal combustion accounted for more than 70% of the total PAHs contribution, which is much greater than the vehicle emission contribution. These results are in accordance with the interpretation of the PAHs isomer ratios.

Compared with the method of PAH isomer ratios, the PCA-MLR method can give quantitative judgment of PAHs sources. However, there may be some limitations for the PCA-MLR method applied to PAHs source apportionment. First, the PCA-MLR method focuses the combination of compound characteristics with higher loads, and based on the monitoring of atmospheric gaseous and particulate PAHs nearby a single emission source, and it requires that the composition characteristics of the desired compound from the emission source to the environmental receptors did not change [44, 45]. However, the composition characteristics of the PAHs compound may change due to some complex environmental processes from the emission source into the atmosphere. Second, the use of PCA for the main component extraction may not be able to distinguish effectively these sources, thus reducing the resolution and accuracy of the PCA-MLR method, since the PAHs compositions in the atmosphere are often a mixture of various emission sources. Third, The PCA-MLR method uses the findings from the source emission characteristics in different regions for PAHs source apportionment, which will bring some uncertainty in the results, since some differences in the PAHs composition in different regions with the same emission sources may exist [4].

### 3.3. Backward Trajectories and Possible Source Regions

**Figure 8:**
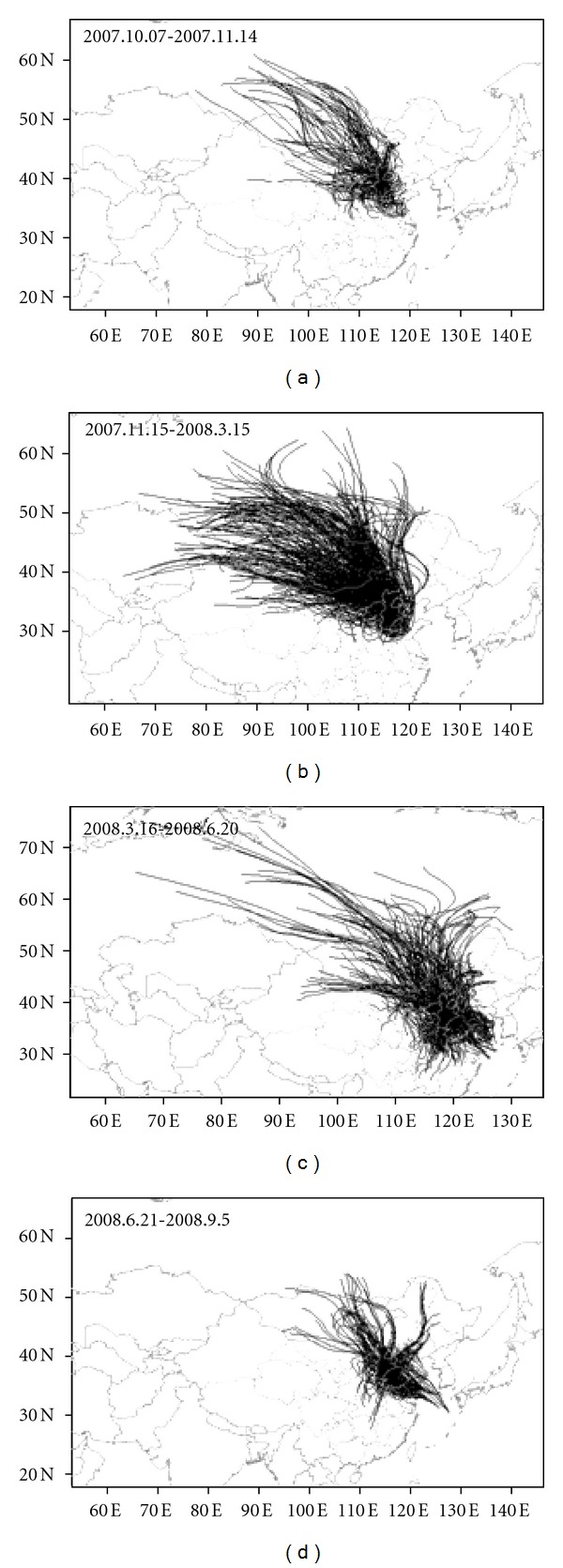
Backward trajectories to Lake Small Baiyangdian in four time.

**Figure 9:**
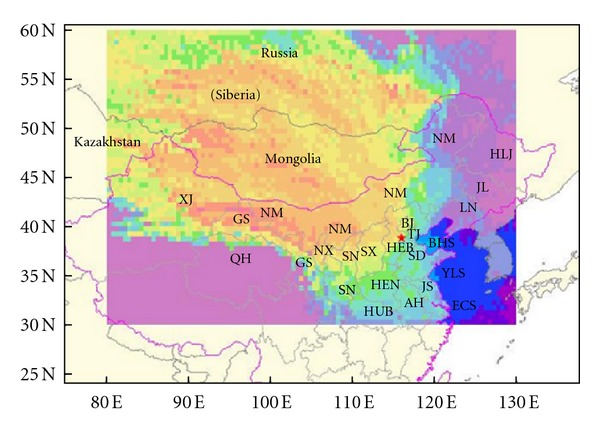
Distribution of possible source regions passed by backward trajectories to Lake Small Baiyangdian in four seasons. HEB : Hebei, HEN : Henan, HUB : Hubei, BJ : Beijing, TJ : Tianjin, SD : Shandong, JS : Jiangsu, AH : Anhui, SX : Shanxi, SN : Shaanxi, NX : Ningxia, GS : Gansu, QH : Qinghai, XJ : Xinjiang, NM : Neimeng, HLJ : Heilongjiang, JL : Jilin, Ln : Liaoning, BHS : Bohai Sea, YLS : Yellow Sea, ECS : East China Sea.

The backward trajectories and possible source regions of air mass are illustrated in Figures 8 and 9, respectively. To further investigate the compositions of the trajectories, cluster analysis was applied, and the results are presented in Figure S1–S6 in the Supplementary Materials available online at doi:10.1100/2012/416321. The process of the cluster number determination and the monthly distribution of each cluster are shown in Figures S1 and S2, respectively. The trajectories in each cluster and the corresponding percentage in the four seasons, autumn, winter, spring, and summer are illustrated in Figures S3–S4, respectively.

In the autumn, the northwest (NNW-NWW) trajectories originated from Siberia and Mongolia (50–60N, 80–110E) were predominant with the percentage of about 31%, followed by the western (NWW-SWW) trajectories from Shanxi, Shaanxi, southwestern Neimeng and Gansu (25%), and by southern (SW-SE) trajectories from Shanxi, Shandong, Tianjin, the northern part of Anhui and Henan (21%). The percentages for the northern trajectories (NNW-NNE) from the middle Neimeng, and for the trajectories from the local and surrounding area, Hebei, Beijing, Tianjing, and southern Neimeng were 10% and 13%, respectively (see Figure 8(a) and Figure S3 for details).

In the winter, the northwest (NNW-NWW) trajectories were predominant with the percentage of about 62%, in which the trajectories originated mainly from Xinjiang and western Mongolia (29%), followed by the trajectories from Siberia and Kazakhstan (18%) and by the trajectories from Shanxi, Shaanxi, Gansu, western Neimeng (15%). The percentages for the northern trajectories (NNW-NE) from Siberia and from the local and surrounding area, Hebei, Beijing, middle Neimeng, and eastern Mongolia were 20% and 18%, respectively (see Figure 8(b) and Figure S4 for details).

In the spring, the northwest (NNW-NWW) trajectories were predominant with the percentage of about 31%, in which the trajectories originated mainly from Siberia (19%), followed by the trajectories from Mongolia, Xinjiang, Neimeng, Gansu, Shanxi, and Shaanxi (12%). The percentage for the northern (NWW-NE) trajectories from Jinlin and the eastern part of Siberia, Mongolia, and Neimeng was 18%. The total percentage for the southern (SWW-SEE) trajectories was about 34%, in which the southwestern and southern (SWW-SSE) trajectories from Henan and the southern part of Shanxi and Shaanxi were predominant (22%), followed by the southeastern (SSE-SEE) trajectories from Shandong, Jiangsu, Anhui, and Yellow Sea (12%). The percentage for the trajectories from the local and surrounding area, Hebei, Beijing, and Tianjing was only about 7% (see Figure 8(c) and Figure S5 for details).

In the summer, the southeastern (SSE-SEE) trajectories from Shandong, Jiangsu, Anhui, Yellow Sea, and East Chinese Sea were predominant with the percentage of about 40%. The percentage for the southwestern (SSW-SWW) trajectories from Henan, Hubei, Hunan, and the southern part of Shanxi and Shaanxi was about 15%. The total percentage for the southern (SEE-SWW) trajectories including the southeastern (SSE-SEE) and southwestern (SSW-SWW) ones was 55%. However, the total percentage for the northern (NEE-NWW) trajectories was only about 32%, in which 17% was for the northern-northwestern (NNE-NWW) trajectories from Mongolia, Neimeng, and the adjacent area of Mongolia and Siberia and 15% for the northeastern (NNW-NEE) trajectories from Liaoning, Beijing, and the adjacent area of Mongolian with Heilongjiang, Jilin, and Liaoning. The percentage for the trajectories from the local and surrounding area, Hebei, Beijing and Tianjing was about 13% (see Figure 8(d) and Figure S6 for details).

It can be found through previous analysis that the directions and percentages of the trajectories and the regions passed by backward trajectories to Lake Small Baiyangdian in four seasons were different (Figure 8 and Figure S3–S6). From autumn to winter, the percentages for the northwestern (NNW-NWW) and northern (NNW-NNE) trajectories were increased from 31% and 10% to 62% and 38%, respectively; however, these for the southern (SW-SE) and western (SWW-NWW) trajectories were decreased from 21% and 25% to nearly zero. Such changes in the directions and percentages of the trajectories from autumn to winter were caused by the conversion of the summer monsoon with higher percentage of southern air mass to the winter monsoon with higher percentage of northern air mass in the Eastern Asia [46]. From winter to spring and to summer, the percentages for the northwestern (NNW-NWW) and northern (NNW-NNE) trajectories were decreased from 62% and 38% in winter to 31% and 18% in spring and to 17% and 15% in summer. However, the percentages for the southern (SWW-SEE) trajectories were increased from nearly zero in winter to 34% in spring and to 55% in summer. Such changes in the directions and percentages of the trajectories winter to spring and to summer were caused by the gradual conversion of the winter monsoon with higher percentage of northern air mass to the summer monsoon with higher percentage of southern air mass to the Eastern Asia [46].

The source regions of the backward trajectories and the distances from the source regions to Lake Small Baiyangdian in four seasons were also different (Figures 8 and 9 and Figures S3–S6). For northwestern trajectories caused by winter monsoon, the yearly dominant air mass, their effected distances in winter and spring are much longer than these in autumn and summer. However, for southeastern trajectories caused by summer monsoon, their effected distances in summer are longer than these in autumn, spring, and winter. The long-distance northwestern source regions of Siberia, Kazakhstan, Mongolia, and Xinjiang were caused by the strong northwestern air mass in winter and spring. The relative short-distance southwestern and southeastern source regions of Henan, Hunan, Anhui, Shandong, Jiangsu, Yellow Sea, and Eastern Chinese Sea were caused by the weak southwestern and southeastern air mass in summer.

## 4. Conclusion

The residual levels, distribution, and partition of PAHs in the gas phase, particulate phase, and in dust fall were studied; the major sources were identified, and the backward trajectories of the air masses for the entire year were calculated. It could be concluded that the PAH16 residual levels in the seasonal gas-phase and particle-phase contents exhibited the following order from high to low values: winter > autumn > spring > summer. The seasonal average concentrations in the dust fall from high to low were winter > autumn > summer > spring. Vehicle emission, coal combustion, and biomass combustion were the major sources of PAHs for the Lake Small Baiyangdian atmosphere. The backward trajectories air masses starting from the center of Lake Small Baiyangdian were dominated by the northwesterly air mass pathways. The long-distance northwestern source regions of Siberia, Kazakhstan, Mongolia, and Xinjiang were caused by the strong northwestern air mass in winter and spring. The relative short-distance southwestern and southeastern source regions of Henan, Hunan, Anhui, Shandong, Jiangsu, Yellow Sea, and Eastern Chinese Sea were caused by the weak southwestern and southeastern air mass in summer.

## Supplementary Material

The supplementary materials include six figures. The titles for these figures are as follows:
Fig. S1 Values of R^2^ as a function of the number of clusters for the backward trajectories at Lake Small Baiyangdian in four time periods. The black arrow in the four figures from (a)–(d) points to the chosen cluster numbers in the first stage. The grey arrows in figure (c) point to the chosen cluster numbers of the short-trajectory groups in the second stage. The chosen number was the number before a large change in the corresponding R^2^ values could be observed as the cluster number decreased.
Fig. S2 Monthly distributions of the trajectories in the clusters determined by the k-means-based clustering method in the four time periods.
Fig. S3 The trajectories in the clusters determined by the k-means-based clustering method in time period ‘a' from 2007.10.07 to 2007.11.14 and the corresponding percentage.
Fig. S4 The trajectories in the clusters determined by the k-means-based clustering method in time period ‘b' from 2007.11.15 to 2008.03.15 and the corresponding percentage.
Fig. S5 The trajectories in the clusters determined by the k-means-based clustering method in time period ‘c' from 2008.03.16 to 2008.06.20 and the corresponding percentage.
Fig. S6 The trajectories in the clusters determined by the k-means-based clustering method in time period ‘d' from 2008.06.21 to 2008.09.05 and the corresponding percentage.

## Figures and Tables

**Table 1:** Recoveries and instrumental detection limits.

PAHs	Recoveries			Instrumental detection limits (ng·mL^−1^)
	Gas	Particle	Dust fall
NAP	46%	47%	56%	1.02
ACE	51%	48%	71%	0.76
ACY	67%	50%	77%	0.79
FLO	75%	57%	85%	0.87
PHE	83%	69%	83%	1.8
ANT	77%	71%	87%	0.64
FLA	98%	87%	98%	0.85
PYR	124%	88%	104%	0.8
BaA	99%	97%	99%	0.9
CHR	92%	102%	92%	1.2
BbF	121%	103%	121%	1.85
BkF	90%	111%	90%	1.1
BaP	108%	103%	108%	0.85
DahA	102%	119%	102%	1.52
IcdP	127%	118%	120%	1.8
BghiP	65%	115%	75%	1.38

**Table 2:** PAHs contents in the gaseous phase, particulate phase, and dust fall at Lake Small Baiyangdian.

	Gaseous phase (ng·m^−3^)				Particulate phase (ng·m^−3^)				Dust fall (ng·g^−1^)			
	Mean	SD	Min	Max	Mean	SD	Min	Max	Mean	SD	Min	Max
Nap	123.4	157.2	7.7	478.1	7.7	4.7	2.4	16.7	151.7	74.1	33.4	254.8
Ace	7.6	6.1	1.3	16.7	0.3	0.2	0.1	0.8	16.1	16.4	5.8	52.5
Acy	3.1	3.7	0.2	9.4	0.8	0.8	0.0	2.2	31.8	22.0	7.6	64.4
Flo	66.9	49.9	16.1	161.4	3.5	2.5	1.4	8.2	132.5	79.4	58.8	297.7
Phe	142.6	130.0	42.4	428.0	21.1	21.5	4.0	71.2	1553.5	1167.0	412.1	3738.6
Ant	6.5	5.9	1.6	19.1	2.1	1.7	0.9	5.0	121.6	105.8	36.7	350.1
Fla	39.9	36.7	6.6	120.2	22.3	20.1	4.2	64.9	1471.1	1149.9	391.8	3676.1
Pyr	19.5	18.4	3.9	60.5	12.6	10.3	3.5	34.7	796.9	579.4	232.9	1934.6
Baa	1.2	1.1	0.3	3.7	9.2	2.1	5.4	11.1	210.8	69.3	116.3	324.2
Chr	3.1	2.3	0.8	7.9	17.1	4.6	9.4	21.9	661.1	274.2	334.5	1140.4
Bbf	1.8	2.0	0.3	5.6	19.5	2.5	15.0	21.9	756.2	273.2	411.9	1162.2
Bkf	0.6	0.6	0.2	1.4	14.4	3.0	9.4	17.3	225.2	69.1	129.2	323.3
Bap	0.4	0.3	0.1	0.8	13.9	1.0	12.6	15.6	230.8	120.0	122.6	452.2
DahA	0.1	0.0	0.1	0.1	ND	ND	ND	ND	53.9	45.7	18.7	150.7
IcdP	1.0	0.5	0.2	1.4	20.7	0.5	20.0	21.1	302.9	186.1	29.8	556.4
BghiP	0.7	0.5	0.2	1.5	20.7	0.9	19.4	22.0	214.1	99.7	108.4	372.0

LMW	390.0	284.8	76.9	851.6	57.0	47.9	15.8	163.2	3478.3	2278.0	1156.9	6643.5
MMW	26.0	23.0	6.7	74.5	57.7	37.1	3.5	101.2	2650.2	1136.3	1277.5	4884.7
HMW	2.0	1.3	0.6	3.5	50.8	10.0	35.9	57.3	801.7	374.4	429.1	1446.7
PAH16	417.2	299.8	84.4	928.6	150.9	99.2	19.3	321.6	6930.2	3206.5	2916.9	12387.2

Nap: naphthalene; Ace: acenaphthene; Acy: acenaphthylene; Flo: fluorene; Phe: phenanthrene; Ant: anthracene; Fla: fluoranthene; Pyr: pyrene; Baa: benzo[a]anthracene; Chr: chrysene; Bbf: benzo[b]fluoranthene; Bkf: benzo[k]fluoranthene; Bap: benzo[a]pyrene; IcdP: indeno[1,2,3-cd]pyrene; DahA: dibenz[a,h]anthracene; BghiP: benzo[ghi]perylene; PAH16: the sum of 16 PAH components; LMW-PAH: low molecular weight PAHs including 2-3 ring PAHs (Nap, Ace, Acy, Flo, Phe, Ant, Fla); MMW-PAH: moderate molecular weight PAHs including 4 ring PAHs (Pyr, Baa, Chr, Bbf, Bkf); HMW-PAH: high molecular weight PAHs including 5-6 ring PAHs (Bap, Icdp, Daha, Bghip).

ND: not detected.

**Table 3:** Rotated component matrix of PAHs in the gaseous and particulate phases at Lake Small Baiyangdian.

Principal components	1	2	3
Nap	0.275	−0.038	0.896
Acy	0.345	0.443	0.796
Ace	0.379	0.028	0.896
Flo	0.348	0.892	0.242
Phe	0.265	0.963	0.012
Ant	0.165	0.971	0.14
Fla	0.369	0.917	−0.069
Pyr	0.354	0.919	−0.069
Baa	0.77	0.54	0.317
Chr	0.852	0.332	0.352
Bbf	0.915	0.331	0.226
Bkf	0.853	0.338	0.397
Bap	0.923	0.336	0.159
Icdp	0.616	0.411	−0.615
BghiP	0.948	0.205	0.221

Estimated sources	Vehicle	Coal	Wood
Variance (%)	38.86	36.67	21.42
