# Supplementary material for: Distributions, Sources, and Backward Trajectories of Atmospheric Polycyclic Aromatic Hydrocarbons at Lake Small Baiyangdian, Northern China
Source: ScientificWorldJournal. 2012 Oct 15;2012:416321. doi: 10.1100/2012/416321 (PMC3478730; doi:10.1100/2012/416321)
Supplement: Supplementary file 1 — The supplementary materials include six figures. The titles for these figures are as follows: Fig. S1 Values of R2 as a function of the number of clusters for the backward trajectories at Lake Small Baiyangdian in four time periods. The black arrow in the four figures from (a)–(d) points to the chosen cluster numbers in the first stage. The grey arrows in figure (c) point to the chosen cluster numbers of the short-trajectory groups in the second stage. The chosen number was the number before a large change in the corresponding R2 values could be observed as the cluster number decreased. Fig. S2 Monthly distributions of the trajectories in the clusters determined by the k-means-based clustering method in the four time periods. Fig. S3 The trajectories in the clusters determined by the k-means-based clustering method in time period ‘a' from 2007.10.07 to 2007.11.14 and the corresponding percentage. Fig. S4 The trajectories in the clusters determined by the k-means-based clustering method in time period ‘b' from 2007.11.15 to 2008.03.15 and the corresponding percentage. Fig. S5 The trajectories in the clusters determined by the k-means-based clustering method in time period ‘c' from 2008.03.16 to 2008.06.20 and the corresponding percentage. Fig. S6 The trajectories in the clusters determined by the k-means-based clustering method in time period ‘d' from 2008.06.21 to 2008.09.05 and the corresponding percentage. [file 416321.f1.pdf]

Supplementary Materials for

Distributions, sources and backward trajectories of atmospheric polycyclic aromatic hydrocarbons at Lake Small Baiyangdian, northern China

Ning Qin, Xiang-Zhen Kong, Ying Zhu, Wei He, Qi-Shuang He, Bin Yang, Hui-Ling Ou-Yang, Wen-Xiu Liu, Qing-Mei Wang, Fu-Liu Xu*

The supplementary materials include six figures. The titles for these figures are as follows:

Fig. S1 Values of R^2 as a function of the number of clusters for the backward trajectories at Lake Small Baiyangdian in four time periods. The black arrow in the four figures from (a)-(d) points to the chosen cluster numbers in the first stage. The grey arrows in figure (c) point to the chosen cluster numbers of the short-trajectory groups in the second stage. The chosen number was the number before a large change in the corresponding R^2 values could be observed as the cluster number decreased.

Fig. S2 Monthly distributions of the trajectories in the clusters determined by the k-means-based clustering method in the four time periods.

Fig. S3 The trajectories in the clusters determined by the k-means-based clustering method

in time period 'a' from 2007.10.07 to 2007.11.14 and the corresponding percentage.

Fig. S4 The trajectories in the clusters determined by the k-means-based clustering method in time period 'b' from 2007.11.15 to 2008.03.15 and the corresponding percentage.

Fig. S5 The trajectories in the clusters determined by the k-means-based clustering method in time period 'c ' from 2008.03.16 to 2008.06.20 and the corresponding percentage.

Fig. S6 The trajectories in the clusters determined by the k-means-based clustering method in time period 'd' from 2008.06.21 to 2008.09.05 and the corresponding percentage.

* Correspondence author: Fu-Liu Xu, MOE Laboratory for Earth Surface Processes, College of Urban & Environmental Sciences, Peking University, Beijing 100871, China; phone: 00861062751177; e-mail:(xufl@urban.pku.edu.cn).

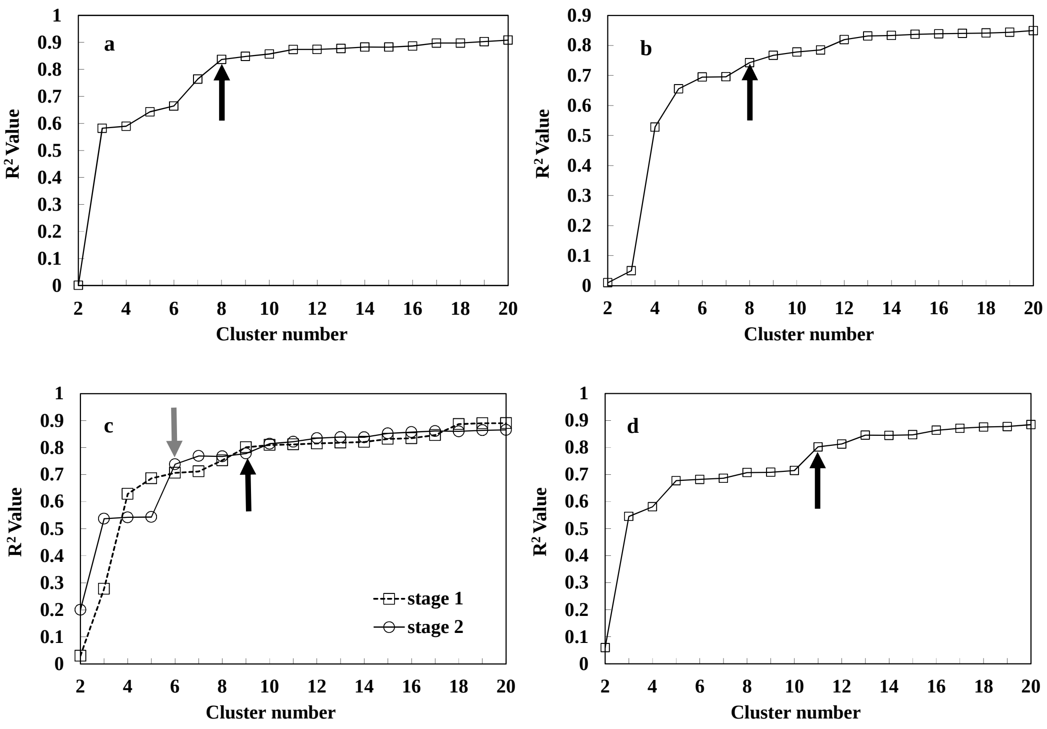

Fig. S1 Values of R^2 as a function of the number of clusters for the backward trajectories at Lake Small Baiyangdian in four time periods. The black arrow in the four figures from (a)-(d) points to the chosen cluster numbers in the first stage. The grey arrows in figure (c) point to the chosen cluster numbers of the short-trajectory groups in the second stage. The chosen number was the number before a large change in the corresponding R^2 values could be observed as the cluster number decreased.

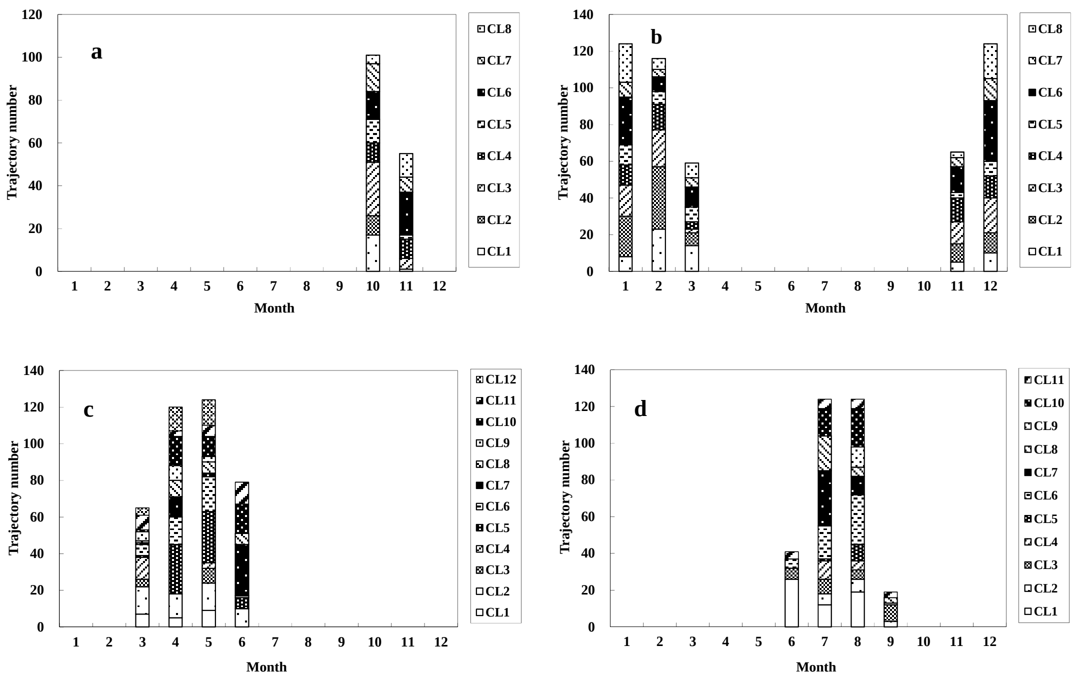

Fig. S2 Monthly distributions of the trajectories in the clusters determined by the k-means-based clustering method in the four time periods.

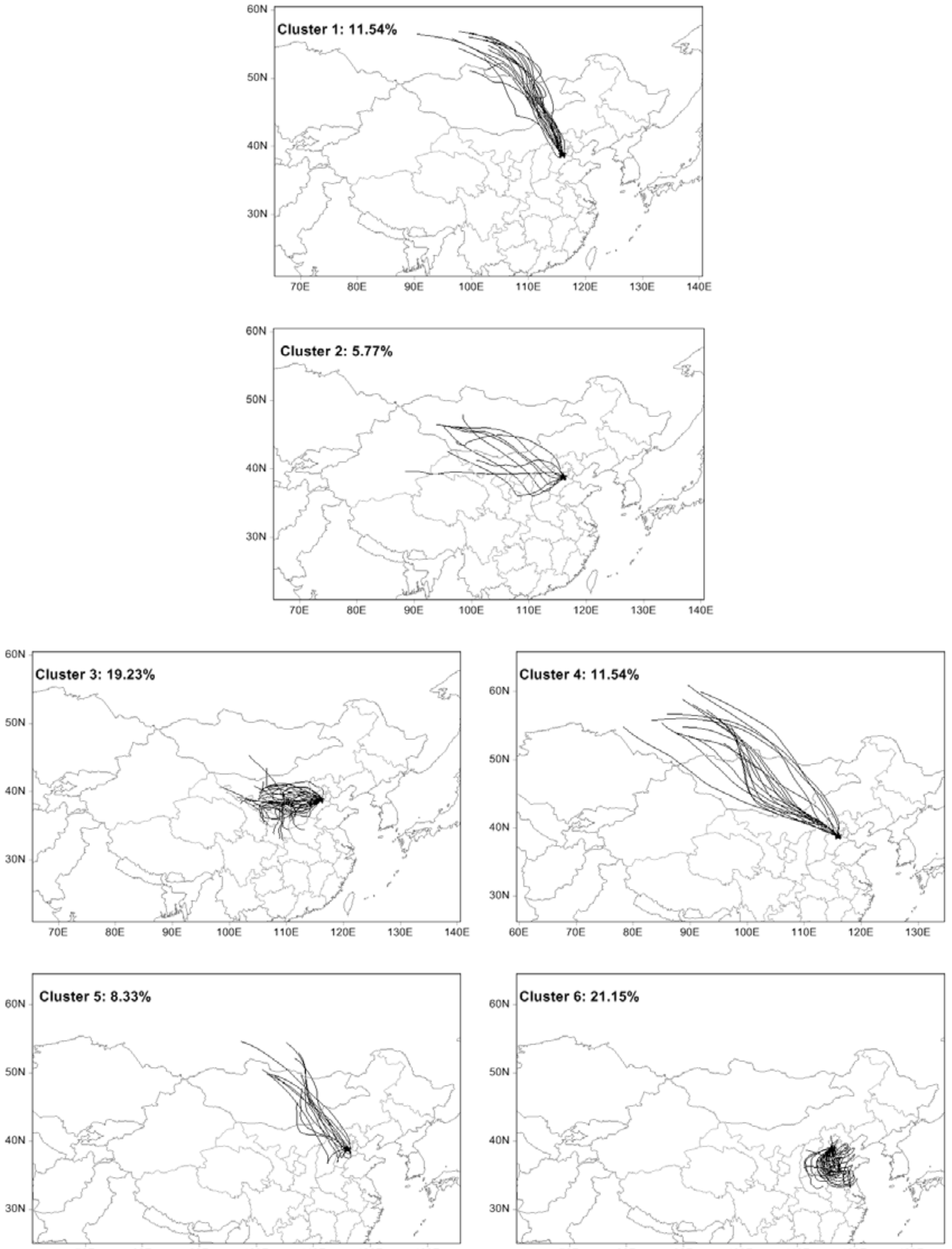
Cluster 1: 11.54%
Cluster 2: 5.77%
Cluster 3: 19.23%
Cluster 4: 11.54%
Cluster 5: 8.33%
Cluster 6: 21.15%
60N
50N
40N
30N
60E
70E
80E
90E
100E
110E
120E
130E
140E

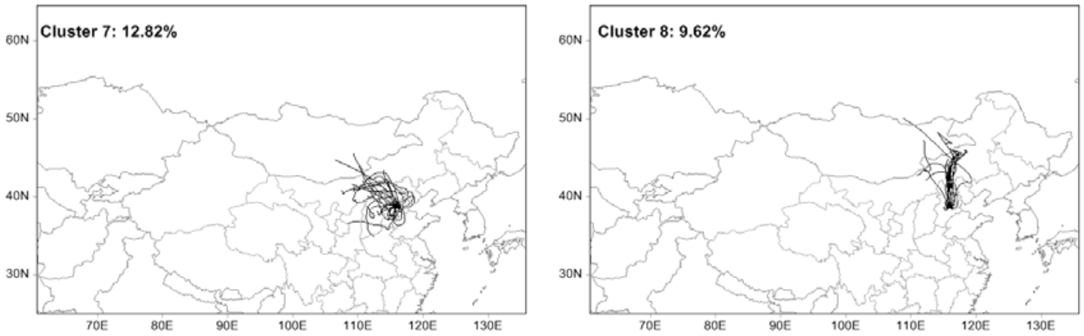

Fig. S3 The trajectories in the clusters determined by the k-means-based clustering method in time period ‘a’ from 2007.10.07 to 2007.11.14 and the corresponding percentage.

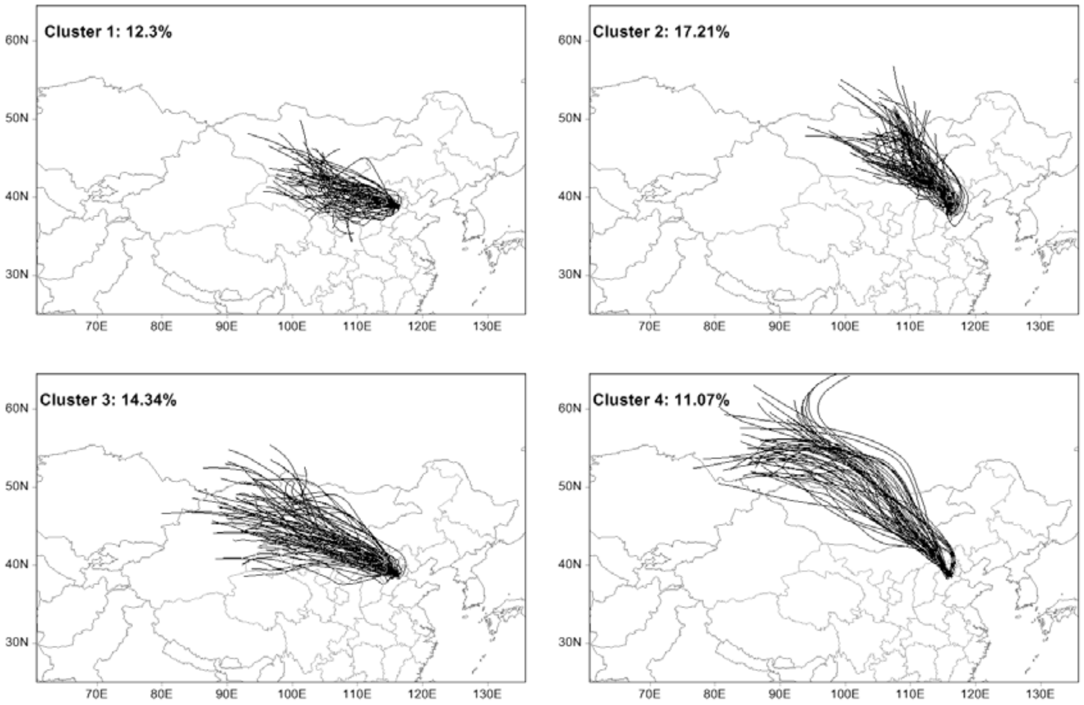

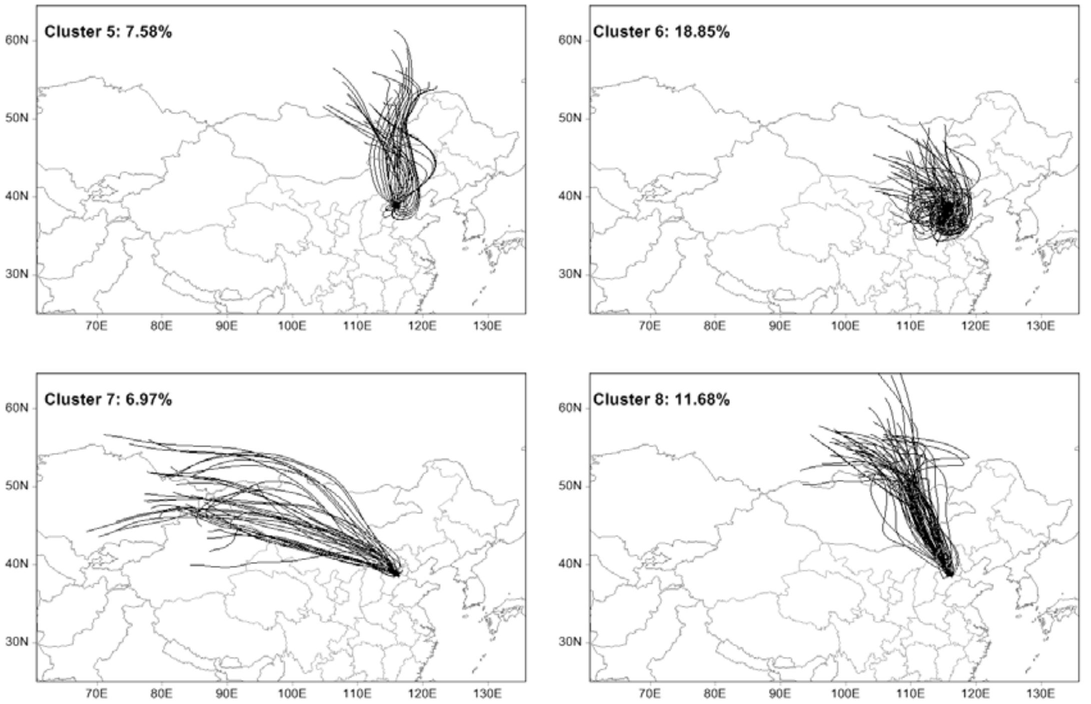

Fig. S4 The trajectories in the clusters determined by the k-means-based clustering method in time period ‘b’ from 2007.11.15 to 2008.03.15 and the corresponding percentage.

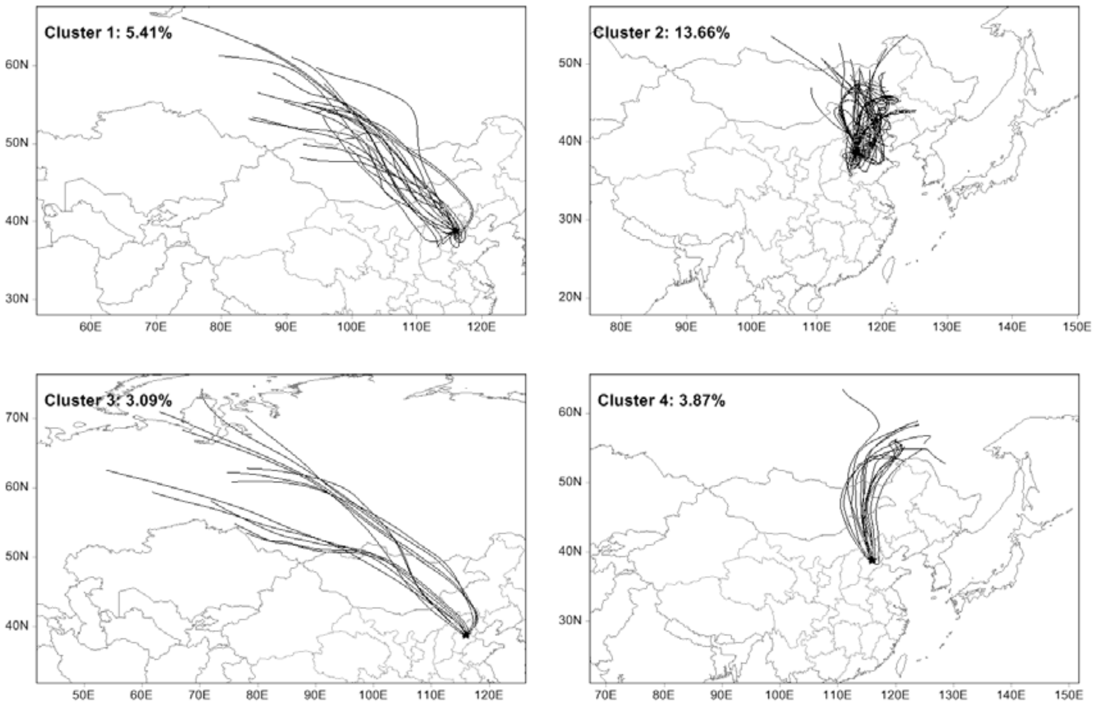

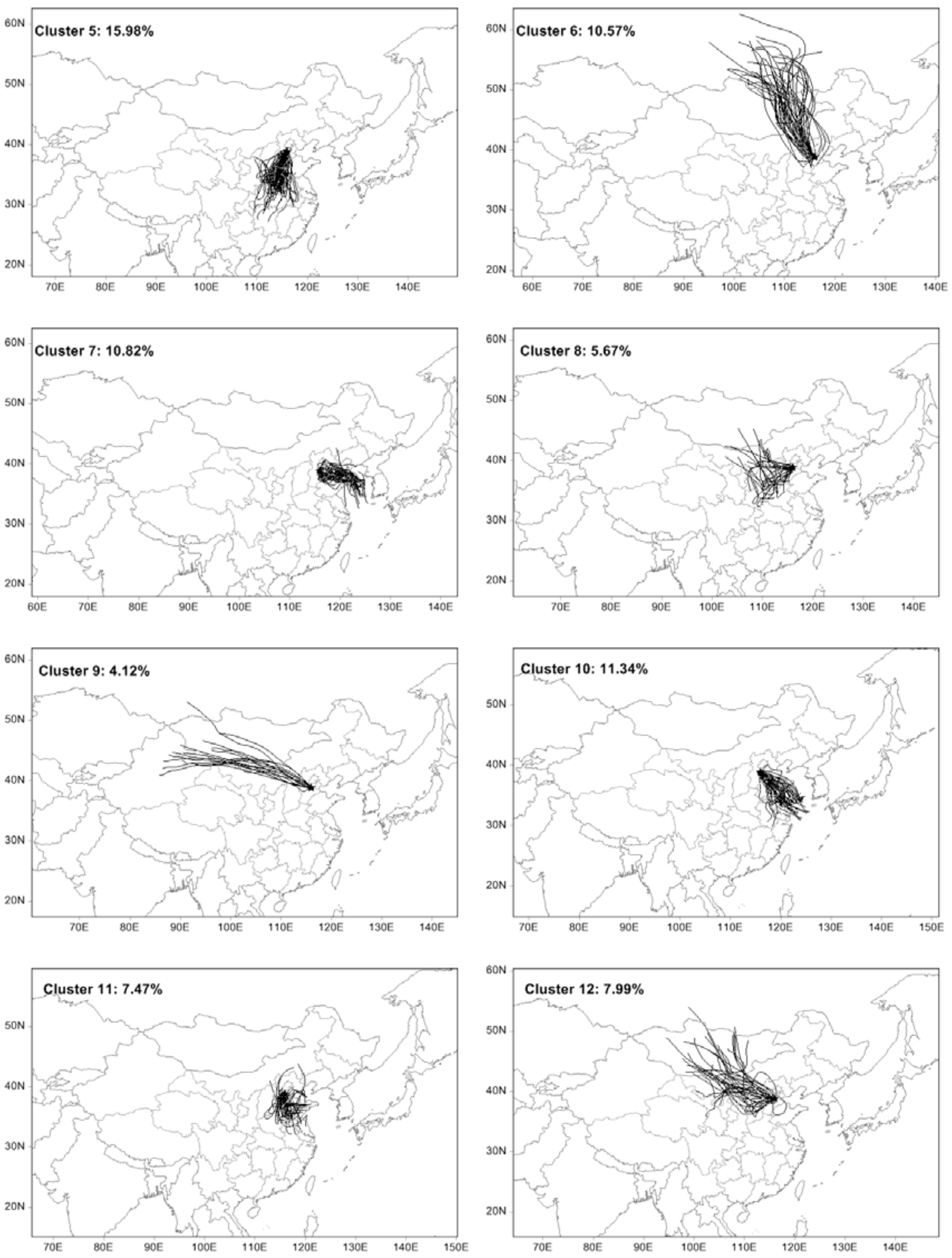

Fig. S5 The trajectories in the clusters determined by the k-means-based clustering method in time period ‘c ’ from 2008.03.16 to 2008.06.20 and the corresponding percentage.

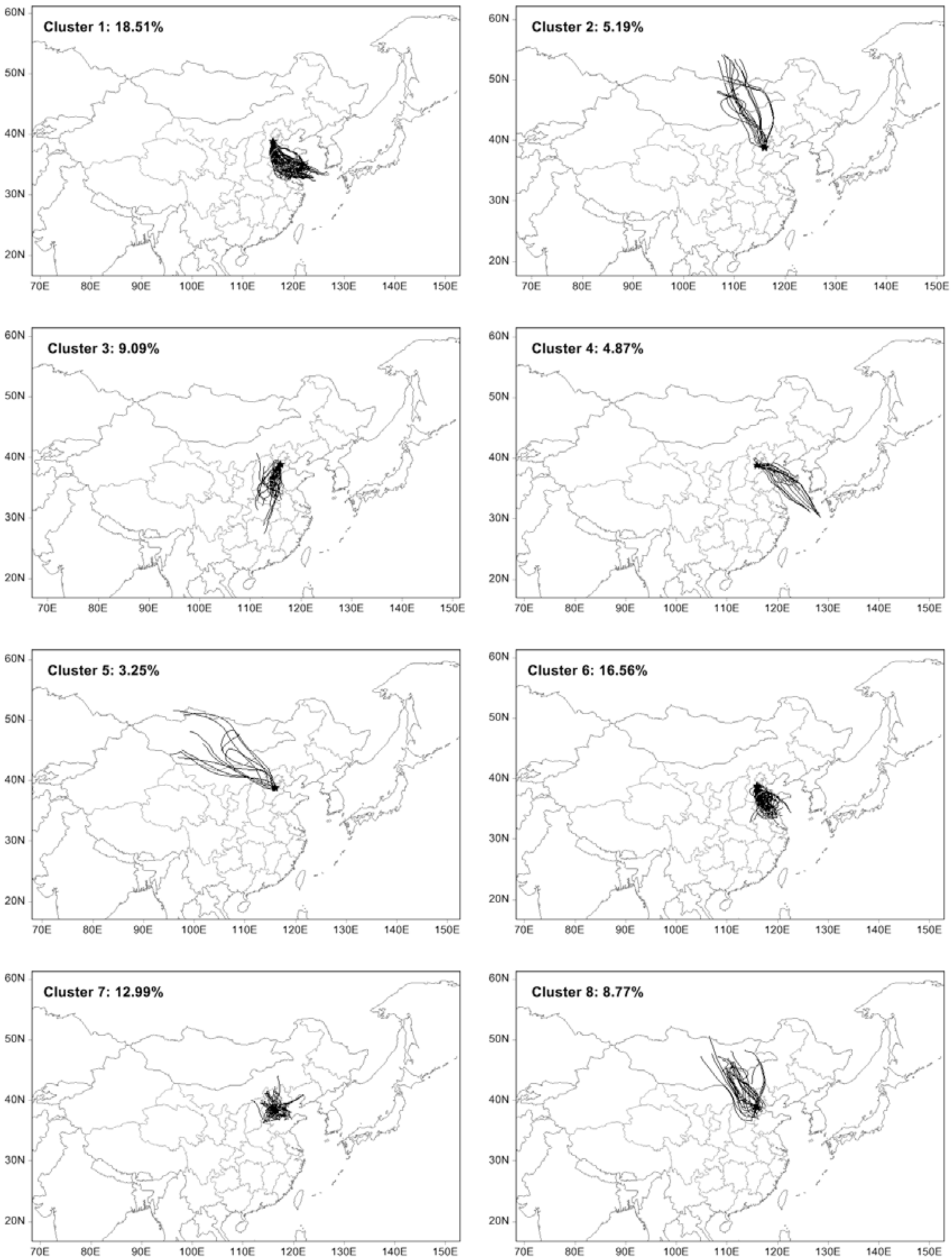
Cluster 1: 18.51%
Cluster 2: 5.19%
Cluster 3: 9.09%
Cluster 4: 4.87%
Cluster 5: 3.25%
Cluster 6: 16.56%
Cluster 7: 12.99%
Cluster 8: 8.77%
60N
50N
40N
30N
20N
70E
80E
90E
100E
110E
120E
130E
140E
150E

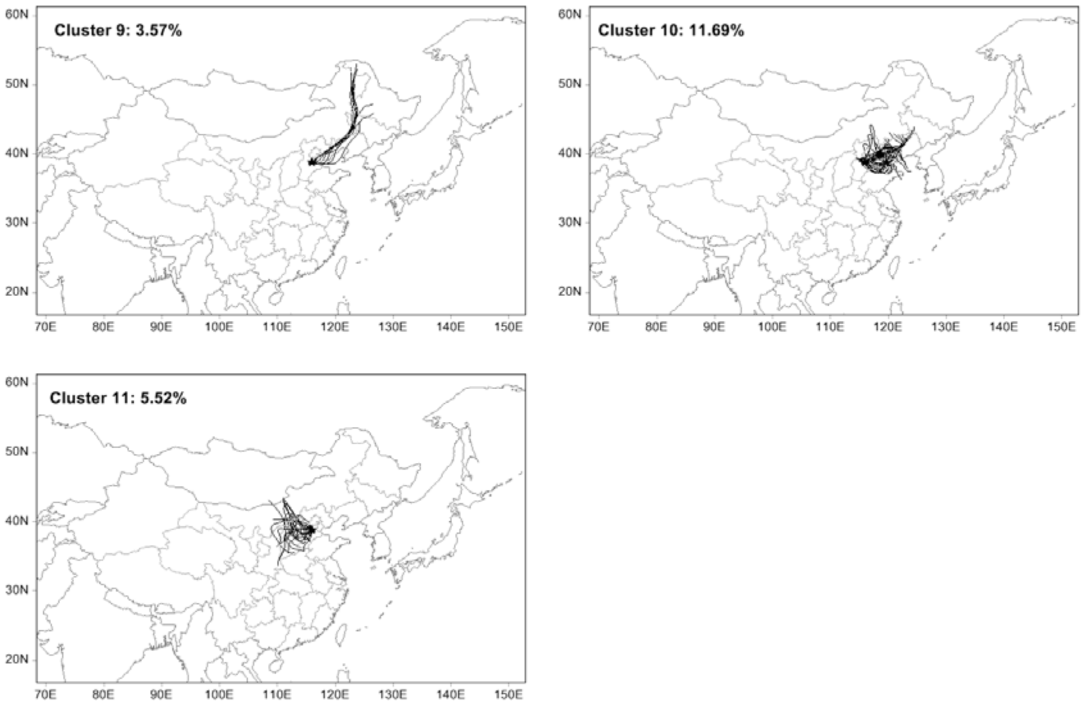

Fig. S6 The trajectories in the clusters determined by the k-means-based clustering method in time period ‘d’ from 2008.06.21 to 2008.09.05 and the corresponding percentage.
